# Approach to Diagnosis of TFE3-rearranged Renal Cell Carcinoma in a Limited Resource Setting: A Case Report

**DOI:** 10.15586/jkcvhl.v11i3.338

**Published:** 2024-08-24

**Authors:** Allison Kaye Lombridas Pagarigan, Pamela Delos Reyes-Murillo, Dennis Jose Sienes Carbonell

**Affiliations:** Department of Pathology and Laboratory Medicine, National Kidney and Transplant Institute, East Avenue, Barangay Central, Quezon City, Philippines

**Keywords:** immunohistochemistry, kidney tumor, renal cell carcinoma, TFE3 rearrangement, Xp11.2 translocation

## Abstract

This report recounts the diagnostic workup of a pediatric female who presented with hematuria secondary to a large renal mass visualized on abdominal imaging. Histologic assessment and subsequent immunohistochemistry studies were performed. Intense, unequivocal immunohistochemical expression of TFE3 and alpha-methylacyl-CoA-racemase with corresponding negativity for carbonic anhydrase IX, along with highly distinctive clinical, radiologic, gross, and microscopic findings confirmed the diagnosis of a renal cell carcinoma with TFE3 gene rearrangement – the first ever reported case in the Philippines. This case highlights the vital role and significant diagnostic impact of reliable, affordable and accessible immunohistochemistry studies in low-resource settings where molecular modalities for evaluating rare diseases are largely unavailable. Recognition of distinctive morphologic, immunohistochemical, and cytogenetic features in childhood and adolescent renal malignancies allows for the timely institution of therapeutic interventions for this aggressive entity.

## Introduction

Renal cell carcinoma (RCC) accounts for just 1–5% of renal malignancies in children and adolescents. Of these, 40% are diagnostically classified as chromosome Xp11.2 translocation RCC with transcription factor binding to IGHM enhancer 3 (TFE3) gene rearrangement. This entity has recently been recognized as a molecularly defined category of RCCs in the 2022 World Health Organization (WHO) classification of tumors of the kidney following increasing reports in the literature. Falling under the umbrella of the microphthalmia transcription factor (MiT) family of RCCs, along with transcription factor EB (TFEB) rearranged RCC, overexpression of TFE3 results in the activation of downstream targets physiologically influenced by the MiT transcription factor family. This unique pathogenesis leads to a peculiar and distinctive immunomorphologic presentation of the disease, characterized by the presence of pigmented cells and melanocytic marker expression in microscopy (1). The development of the TFE3 immunohistochemistry antibody reagent has been pivotal for the timely workup of suspected cases of this rare and aggressive disease, which has traditionally been confirmed by gene sequencing and fluorescence in situ hybridization (FISH). Detailed in this paper is the first ever reported case of TFE3-rearranged RCC in the Philippines, with a diagnosis established using a select panel of immunohistochemical markers interpreted in correlation with classic morphological, radiologic, and clinical features.

## Case Report

**Figure 1:**
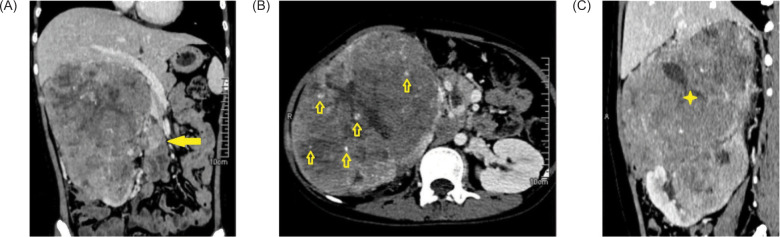
Contrast-enhanced CT-scan of the whole abdomen. (A) Renal mass with renal vein thrombosis (arrow). (B) Intratumoral calcifications (arrows). (C) Large heterogeneously-enhancing mass (star) arising from the kidney.

A 19-year-old female presented with a clinical history of dysuria and a progressively enlarging, eventually palpable right flank mass. A computed tomography (CT) scan of the abdomen (Figure 1) revealed a large, lobulated, heterogeneously-enhancing renal mass (Figure 1C) with internal calcifications (Figure 1B) and hypodense necrotic areas. Thrombosis of the right renal vein and inferior vena cava were also detected (Figure 1A).

A concurrent chest CT scan performed for metastatic workup showed several subcentimeter, non-calcified pulmonary and pleural-based nodules in the posterior segment of the right upper lobe and the superior segment of the right lower lobe. There was no evidence of pleural effusion or enlarged mediastinal lymph nodes.

Anti-hilar sectioning of the nephrectomy specimen revealed a 20.3 x 14.4 x 11.7 cm^3^ ill-defined, irregularly-shaped, firm to friable, lobulated, yellow to tan-gray, grossly hemorrhagic and necrotic mass almost completely replacing the entire renal parenchyma. A large tumor thrombus was identified within the renal vein.Microscopic sections revealed a tumor with predominantly lobulated, nested, and fused papillary architecture, characterized by central areas of necrosis bordered by thin hyalinized and vascularized septa (Figure 3A). The neoplastic cells exhibited moderate to marked pleomorphic, with vesicular nuclei, occasional multinucleate forms, prominent nucleoli, abundant pale eosinophilic to clear cytoplasm, and fairly distinct cell borders (Figure 3B). Several psammoma bodies (Figure 3C) and scattered foci of tumor cells containing melanin pigments (Figure 3D) were identified. Microscopic involvement of the capsule and renal sinus was identified. Excluding the renal vein, all other hilar structures were negative for tumor.

**Figure 2:**
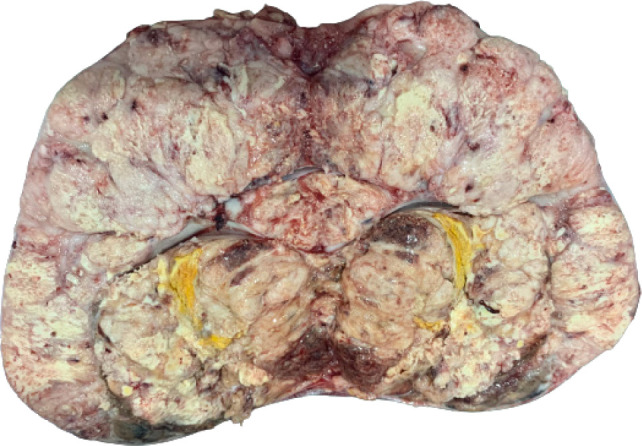
Bivalved nephrectomy specimen revealing a large, lobulated mass.

**Figure 3:**
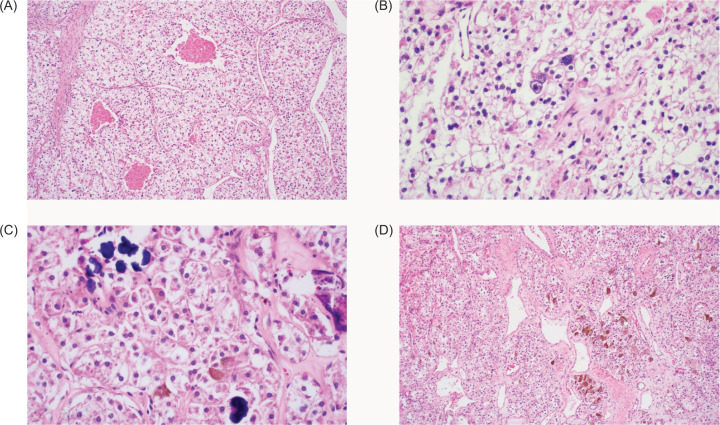
Photomicrographs of hematoxylin and eosin-stained sections exhibiting: (A) variable tumor architecture and necrosis (40×), (B) WHO/ISUP Grade 4 nuclei and cytoplasmic clearing (100×), (C) psammoma bodies (100×), (D) melanin pigments (40×).

**Figure 4:**
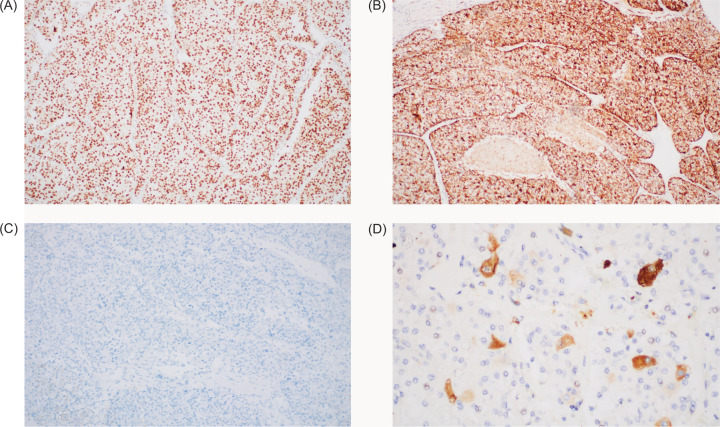
Immunohistochemistry studies. (A) Diffuse nuclear expression of TFE3 (40×). (B) Diffuse positivity for AMACR (40×). (C) Negative CA IX expression (40×). (D) Focal cytoplasmic granular Melan-A expression after bleaching (100×). *TFE3*, transcription factor binding to IGHM enhancer 3. *AMACR*, alpha-methylacyl-CoA racemase.

The described morphologic findings, in relation to the patient’s young age, raised the suspicion for translocation-type renal cell carcinoma, with TFE3-rearranged RCC being the most prevalent pediatric RCC subtype. Immunohistochemistry revealed diffusely positive expression of TFE3 (Figure 4A) and alpha-methylacyl-CoA-racemase (AMACR) (Figure 4B) in almost all tumor cells. Negativity for carbonic anhydrase IX (Figure 4C), effectively ruled out the differential diagnosis of clear cell renal cell carcinoma. Following a bleaching protocol, occasional melan-A expression in neoplastic cells was observed (Figure 4D). A final immunomorphologic diagnosis of TFE3-rearranged renal cell carcinoma was rendered.

## Ethics Statement

Written informed consent was obtained for this retrospective report. This manuscript is registered with the Research Ethics Committee of the Clinical Trial and Research Unit of the National Kidney and Transplant Institute. Institutional review board approval was waived.

## Discussion

As the field of histopathologic diagnosis advances toward establishing molecularly-defined classifications of disease entities, what was once recognized as MiT family translocation renal cell carcinomas has now been further specified into distinct genetic rearrangements involving either the TFE3 or TFEB gene (2). The most recent data from the WHO International Agency for Research on Cancer identifies TFE3-rearranged RCC to be the most common childhood RCC present in as high as 40% of cases. Although grossly variable in appearance, the presence of multiple calcifications in the context of a pediatric renal mass on imaging may alert the clinicians to the possible involvement of this malignancy (1). TFE3-rearranged RCCs are known to manifest as advanced tumors with aggressive behavior, hence the challenge of early recognition and timely intervention (2).

The classic histology of these tumors shows papillary structures lined by epithelioid clear cells with scattered foci of lamellated calcifications. Mixed architectures, such as lobular, nested or tubulocystic patterns, make this disease a great mimicker of other more common RCCs. Peculiar to this entity, however, is the striking presence of numerous psammoma bodies and melanin-pigmented cells, as clearly illustrated in this case (1–2). A survey conducted by Akgul, et.al. showed that among 50 genitourinary pathology specialists who have encountered this rare malignancy, papillary tumor architecture, epithelioid cytology, and abundant clear to eosinophilic cytoplasm were the most consistent microscopic findings (3).

Given the morphologically diverse features of TFE3-rearranged RCC, demonstration of its characteristic immunohistochemical profile is required to establish the diagnosis. Strong and diffuse nuclear immunoreactivity against the C-terminal portion of TFE3 in a clean background is considered highly sensitive and specific for TFE3-rearranged RCC (2). Some specialists consider strong TFE3 staining intensity in more than 75% of tumor cells as diagnostic (3). Positivity for AMACR, pancytokeratin, epithelial membrane antigen and cathepsin K may be used to supplement the diagnosis. Interestingly, up to 22% of cases may express the melanocytic marker melan-A, with rarer expression seen of human melanoma black (HMB-45.) Pertinent negative staining for carbonic anhydrase IX, cytokeratin 7, and PAX8 is observed (1-4). Experts prefer a panel consisting of TFE3, cathepsin K, melan-A, HMB-45, cytokeratin 7 and carbonic anhydrase IX for a comprehensive immunohistochemical work up for suspected cases of TFE3-rearranged RCC (3).

The molecular basis for this malignancy lies in harbored fusion of the TFE3 transcription factor gene located on chromosome Xp11.23 with a rather heterogenous set of fusion partners. The most common fusions involve t(X;1)(p11.2;q21), t(X;17)(p11.2;q25), and t(X;1)(p11.2;p34), corresponding to PRCC, ASPSCR1, and SFPQ gene translocations, respectively (1). The resultant overexpression of TFE3 fusion proteins aberrantly activates the expression of several downstream targets, including those activated by the MiT family of transcription factors, which explains the immunoreactivity to melanocytic markers and cathepsin K (1,4).

The standard for diagnosing this entity remains the demonstration of cytogenetic fusions using FISH break-apart probes. A cut-off of 10% translocation probe positivity is generally accepted, despite the lack of consensus guidelines (3). A comparative study assessing the immunohistochemistry against FISH as the standard for detecting TFE3 gene rearrangement revealed variable sensitivities (70% to 85%) and specificities (57% to 95%) across laboratories, thus concluding that technical differences and lack of standardization tend to undermine the diagnostic utility of immunohistochemistry, making FISH the more consistent confirmatory tool (5). However, an important limitation of FISH is its inability to detect TFE3-rearranged RCCs arising from paracentric inversions of the short arm of chromosome X. In such cases, TFE3 immunohistochemistry, along with confirmatory RNA sequencing or reverse transcription polymerase chain reaction proves to be the superior modality (4).

Given that RCC is fundamentally chemotherapy and radiotherapy resistant, surgery serves to be the cornerstone of treatment for TFE3-rearranged RCC with modern literature citing the utility of targeted chemotherapy and checkpoint inhibitors (6). The outcome of pediatric patients remains guarded with older age at diagnosis being a negative prognostic indicator (1). Pre-operative radiologic tumor assessment using fluorine-18-labelled fluorodeoxyglucose positron emission tomography on two subsequently histologically-confirmed cases showed high standardized uptake values comparable to high-grade RCC. These findings imply tumor aggressiveness and high metastatic potential (7). A recent survival analysis of RCC patients with TFE3 overexpression by FISH and immunohistochemistry showed that tumor recurrence and new metastasis were relatively common even at early stages of the disease. This significantly decreased overall survival is moreover correlated with larger tumor diameter, and presence of lymphatic and vascular invasion in the same study (8). Four months from time of diagnosis, without any instituted adjuvant medical oncologic intervention, the patient presented with bilateral pulmonary metastases detected on chest x-ray. With several poor prognostic indicators including older age at diagnosis, large tumor size and renal vein invasion, she succumbed to her malignancy a month later.

This case largely demonstrates the crucial value of early detection and minimally-invasive diagnostic biopsy procedures that pave the way for timely surgical intervention and initiation of systemic therapies against this aggressive pediatric disease. Careful and extensive tumor sampling of the part of the pathologist is also ideal to determine presence or absence of critical prognostic indicators.

## Conclusion

The diagnosis of TFE3-rearranged RCC rests on recognizing its distinctive morphologic, immunohistochemical, and cytogenetic features within the context of childhood and adolescent renal malignancies. This report demonstrates the diagnostic approach executed in a low-resource setting, where the lack of immunohistochemical markers and more sophisticated molecular modalities for low-prevalence RCC subtypes posed a significant challenge to the encountering pathologists. Resolving the diagnostic dilemma ultimately required the integration of clinical and epidemiologic data, key radiologic findings, judicious gross tumor sampling, a close microscopic evaluation leading to diverse histologic clues, and prioritized immunohistochemical studies that simultaneously targeted confirmatory and pertinent negative expression. The utility of TFE3 immunohistochemistry in RCC can be further optimized through standardization of technical and interpretive protocols and execution of more extensive comparative studies against FISH and other emerging molecular tests. Growing knowledge about the aggressiveness and metastatic potential of TFE3-rearranged RCC only serves to emphasize the need for reliable, affordable and accessible markers that enable pathologists to render timely diagnoses. More definitive studies are needed to determine the optimal approach to managing and prognosticating for this novel classification of kidney tumors.
